# The association between office blood pressure and fluid status using bioimpedance spectroscopy in stable continuous ambulatory peritoneal dialysis patients

**DOI:** 10.1186/s40885-021-00192-0

**Published:** 2022-03-15

**Authors:** Adriaan Slabbert, Mogamat-Yazied Chothia

**Affiliations:** Division of Nephrology, Department of Medicine, Faculty of Medicine and Health Sciences, Stellenbosch University and Tygerberg Hospital, Cape Town, South Africa

**Keywords:** Office blood pressure, Bioimpedance, Peritoneal dialysis, South Africa

## Abstract

**Background:**

Hypertension is common in continuous ambulatory peritoneal dialysis (CAPD) patients. It remains to be determined the extent to which fluid overload contributes to uncontrolled blood pressure (BP) in this population. The aim was to determine the association between fluid status as measured using bioimpedance spectroscopy (BIS) and BP in CAPD patients.

**Methods:**

A cross-sectional study was performed involving 50 stable CAPD patients at a single center in Cape Town, South Africa. All participants were known to have hypertension and were divided into two groups based on office BP measurements: an uncontrolled BP group (systolic BP ≥ 140 mmHg or diastolic BP ≥ 90 mmHg) and a controlled BP group. Fluid status was determined using BIS (Body Composition Monitor®, Fresenius Medical Care, Bad Homburg, Germany).

**Results:**

There was a statistically significant difference in overhydration (OH) between the uncontrolled BP group and the controlled BP group (3.0 ± 2.3 L vs. 1.4 ± 1.6 L, respectively, *P* = 0.01). The uncontrolled BP group was older (37.7 ± 9.5 years vs. 32.0 ± 8.0 years, *P* = 0.04) and had a shorter dialysis vintage (15 [IQR, 7–22] months vs. 31 [IQR, 12–39] months, *P* = 0.02). Significant correlations were found between OH and the extracellular water (ECW) (*r* = 0.557, *P* < 0.01) and ECW to total body water (TBW) ratio (*r* = 0.474, *P* < 0.01). Mixed ancestry, presence of residual kidney function, ECW, and ECW to TBW ratio were identified as predictors of OH on multivariable linear regression.

**Conclusions:**

We found that stable CAPD patients with uncontrolled BP had higher OH compared to patients whose BP was controlled.

## Background

Patients on peritoneal dialysis (PD) frequently present with fluid overload. Depending on the type of hydration parameters used, the reported prevalence of fluid overload ranges from 53.4 to 72.1% [1–3]. The reasons for this may be multifactorial and may include poor dietary salt restriction, particularly following loss of residual kidney function (RKF), less hospital visits, poor glycemic control in patients with diabetes resulting in increased stimulation of the thirst center and reduced ultrafiltration and non-adherence to the prescribed number of daily exchanges. A study that compared hemodialysis and PD reported that overhydration (OH) was significantly higher and occurred more frequently in PD [4].

Optimizing the fluid status in the PD population is critical. Fluid overload in PD patients is associated with increased morbidity and mortality primarily related to cardiovascular disease [5, 6]. Achieving greater small molecule clearance did not result in improved survival in the Canada-USA Peritoneal Dialysis Study Group (CANUSA) and Adequacy of Peritoneal Dialysis in Mexico (ADEMEX) trial [7, 8]. However, a post-hoc analysis of the CANUSA trial found that for every 250 mL of RKF, mortality was reduced by 36% [7] indicating the importance of RKF in the control of fluid status.

The clinical examination has poor diagnostic accuracy with poor reproducibility and high interobserver variation [9]. It was reported that up to a third of patients labelled as being euvolemic by clinical examination were in fact fluid overloaded when assessed by more accurate methods [10]. Using a bioimpedance technique in PD patients, a study found that more than a third were overhydrated in the absence of any clinical signs of fluid overload [11]. As a result, clinicians have searched for alternative methods to accurately assess the volume status in PD patients. These include biomarkers such as atrial and brain natriuretic peptides, inferior vena cava diameter and lung ultrasound [9]. However, bioimpedance techniques have gained popularity in recent years because it is non-invasive, easily applied at the bedside and has been validated against gold standard techniques such as deuterium dilution methods and dual-energy X-ray absorptiometry [12]. Few studies have specifically investigated the association of blood pressure (BP) and fluid status using BIS in the PD population [1, 13, 14]. Even though it is widely accepted that hypertension is associated with an increased intravascular volume [15], measurement of this fluid compartment is difficult, requiring expensive and invasive equipment [16]. Although it was reported that continuous ambulatory peritoneal dialysis (CAPD) patients with uncontrolled hypertension had higher inferior vena cava diameter indices, a marker of intravascular volume, there was no correlation with office BP or ambulatory BP monitoring [17]. However, an association between high BP and expansion of the extracellular fluid compartment has been reported in PD patients [18], as well as an association between arterial stiffness, expansion of the extracellular fluid compartment and uncontrolled hypertension has been described [19].

We aimed to determine the association between fluid status as measured using BIS and office BP in stable CAPD patients.

## Methods

### Study design, population and setting

This was a cross-sectional study of stable CAPD patients at Tygerberg Hospital (TBH) in Cape Town, South Africa. All adult patients (more than 18 years old), with a history of hypertension and a CAPD dialysis vintage of more than 1 month were included. Exclusion criteria included active peritonitis, hemodynamic instability, amputees, current deep vein thrombosis, any metallic intravascular device (such as a pacemaker) or articular prosthesis, pregnancy, automated PD, and patients with current malignancies. The calculated sample size was 58 using a margin of error of 5%, confidence level of 95%, prevalence of fluid overload in PD patients of 50% and a total population size of 67 patients (the latter the total number of PD patients at our center).

This study was approved by the Health Research Ethics Committee (HREC) of Stellenbosch University (HREC study number: S18/02/024) and was performed in accordance with the Declaration of Helsinki.

### Clinical and biological data

After gaining informed consent, participants drained their existing PD fluid dwell and were kept dry during this short period of data collection. Those with RKF emptied their bladders.

The following demographic data were recorded for each patient: age, sex, self-reported ethnicity, dialysis vintage (months), diabetic status, the number and types of antihypertensive drugs and data on the CAPD prescription were collected including the types of PD solutions used and the frequency of daily exchanges. We used self-reporting of RKF, which was defined as an estimated 24-h urine volume of more or equal to than 250 mL (1 cup) since self-reporting of RKF was reported to have a strong agreement with measured urine volumes [20]. The clinically estimated dry weight documented in the patient’s clinic file was recorded and the patient’s weight on the day of data collection was measured using a calibrated, automated scale.

Patients were asked to stop consumption of any foods and beverages containing caffeine at least 30 min before office BP were measured. BP was measured with the participants sitting upright in a chair with the back supported and the arm supported at the level of the heart. In participants with a mid-upper arm circumference of less than or equal to 33 cm, a standard cuff size of 12 cm was used, and a 15 cm cuff was used if the mid-upper arm circumference was more than 33 cm. BP was initially measured in both arms and the remaining BPs were measured in the arm with the highest BP. The initial BP recording was discarded. Patients were divided into two groups based on the average of three BP readings taken at least 1-min apart using a calibrated, automated BP machine (DINAMAP™, GE Medical Systems Information Technologies Inc., Milwaukee, WI, USA). Participants with an average systolic BP of ≥140 mmHg and/or average diastolic BP of ≥90 mmHg were regarded as cases (uncontrolled BP group) while participants with an average systolic BP < 140 mmHg and diastolic BP < 90 mmHg were regarded as controls (controlled BP group).

### Bioimpedance spectroscopy data

The bioimpedance spectroscopy device used to assess the fluid status was the Body Composition Monitor (BCM®), Fresenius Medical Care, Bad Homburg, Germany. Two electrodes were placed on the upper limb (wrist and dorsum of the hand) and two electrodes were placed on the ipsilateral lower limb (ankle and dorsum of the foot). The measurements were taken in a supine position and the average time of completion was 3 min. The following output data were recorded: total body water in liters (TBW), extracellular water in liters (ECW), ECW to TBW ratio, intracellular water in liters (ICW), lean tissue index (LTI) in kilograms per square meter, fat tissue index (FTI) in kilograms per square meter, dry weight in kilograms and OH in liters. Participants resumed PD after BIS was completed.

### Statistical analysis

All continuous variables with a normal distribution were reported as means and standard deviations and those that were not normally distributed were reported as median and interquartile range (IQR). Chi-squared and Fisher’s exact tests were used to compare categorical variables. Mann-Whitney U test was used to compare continuous variables that were not normally distributed while Student t-test was used for normally distributed data. Pairwise correlation was used to determine the correlation between OH and age, dialysis vintage, systolic BP, diastolic BP, mean arterial pressure, TBW, ECW, ICW, ECW to TBW ratio, LTI and FTI. Stepwise forward multiple regression was performed to identify predictors of OH. Statistical significance was regarded as a *P* < 0.05 and 95% confidence intervals were used. Data were analyzed using Stata ver. 16.1 (StataCorp LLC, College Station, TX, USA).

## Results

A total of 67 patients were screened. Seventeen patients were excluded for the following reasons: work-related restrictions (5), temporary modality change (3), inability to gain informed consent (3), peritonitis (2), PD for less than 1-month (2), PD stopped due to partial kidney recovery (1) and APD (1). Fifty patients were included in the final statistical analysis of which 17 (34%) of the patients were in the controlled BP group and 33 (66%) were in the uncontrolled BP group.

Table 1 shows the demographic, clinical and BIS measurements data for the two groups. There was a significant difference in the mean age between groups (32.0 ± 8.0 years vs. 37.7 ± 9.5 years in the controlled BP group and the uncontrolled BP group, respectively; *P* = 0.04). There were no differences in ethnicity or sex. The median PD vintage for the controlled BP group was 31 (interquartile range [IQR], 12–39) months vs. 15 (IQR, 7–22) months in the uncontrolled BP group; *P* = 0.02. There were no differences in self-reported RKF, diabetes mellitus status, the mean or absolute number and types of antihypertensive drugs or the timing of antihypertensive drugs relative to the BIS measurements. There were no differences in the type of PD solutions used or the frequency of exchanges between the two groups. None of the patients were using 4.25% PD solutions, shown in Table 2.

**Table 1:** Baseline demographic, clinical and bioimpedance analysis data

Baseline characteristics	Total (*n* = 50)	Controlled BP group (*n* = 17)	Uncontrolled BP group (*n* = 33)	*P*-value
**Demographic data**				
Age (yr)	35.8 ± 9.4	32.0 ± 8.0	37.7 ± 9.5	0.04
Female sex	28 (56.0)	11 (64.7)	17 (51.5)	0.37
Self-reported ethnicity				
Black	22 (44.0)	7 (41.2)	15 (45.5)	0.77
Mixed ancestry	28 (56.0)	10 (58.8)	18 (54.5)	
**Clinical data**				
Diabetes mellitus	2 (4.0)	1 (5.9)	1 (3.0)	1.00
Dialysis vintage (mo)	21 (8–31)	31 (12–39)	15 (7–22)	0.02
Self-reported RKF (≥250 mL/day)	29 (58.0)	8 (47.1)	21 (63.6)	0.26
Mean no. of antihypertensives	2.7 ± 1.0	2.8 ± 1.2	2.6 ± 0.9	0.54
No. of antihypertensives				
0–2	23 (46.0)	9 (52.9)	14 (42.4)	0.48
≥ 3	27 (54.0)	8 (47.1)	19 (57.6)	
Took antihypertensive drugs on day of BIS measurements: yes	37 (74.0)	12 (70.6)	25 (75.8)	0.74
Type of antihypertensive drugs				
Calcium channel blocker	33 (66.0)	11 (64.7)	22 (66.7)	0.89
Angiotensin-converting enzyme inhibitor	26 (52.0)	9 (52.9)	17 (51.5)	0.76
Angiotensin receptor blocker	9 (18.0)	3 (17.6)	6 (18.2)	1.00
Loop diuretic	38 (76.0)	12 (70.6)	26 (78.8)	0.73
Mineralocorticoid receptor antagonist	1 (2.0)	0	1 (3.0)	–
Alpha blocker	9 (18.0)	4 (23.5)	5 (15.2)	0.47
Non-selective alpha-1 and beta-receptor blocker	14 (28.0)	6 (35.3)	8 (24.2)	0.51
Cardio-selective beta-1 receptor blocker	5 (10.0)	3 (17.6)	2 (6.0)	0.32
Systolic BP (mmHg)	138.8 ± 20.8	116 ± 14.1	150 ± 12.5	< 0.01
Diastolic BP (mmHg)	91.0 ± 13.9	76.5 ± 8.6	98.5 ± 9.4	< 0.01
MAP (mmHg)	106 ± 15.5	89.7 ± 9.9	115.8 ± 9.0	< 0.01
Weight on the day of data collection (kg)	65.2 (57.3–72.0)	66.3 (57.3–72.1)	63.4 (57.7–75.5)	0.99
Clinically estimated dry weight (kg)	65.2 (56.7–70.2)	62.2 (57.9–68.9)	66.5 (56.7–73.7)	0.64
BMI (kg/m^2^)	23.6 (21.6–26.7)	24.3 (21.6–26.7)	23.6 (21.7–26.6)	0.83
**Bioimpedance measurement**				
Dry weight (kg)	62.7 (55.2–71.3)	66.0 (55.2–71.1)	62.0 (56.2–71.4)	0.91
TBW (L)	34.8 ± 6.7	33.0 ± 6.4	35.7 ± 6.8	0.18
ECW (L)	16.5 ± 3.3	15.3 ± 2.9	17.1 ± 3.4	0.07
ECW to TBW	0.48 ± 0.04	0.47 ± 0.04	0.48 ± 0.05	0.49
ICW (L)	18.2 ± 4.0	17.6 ± 3.9	18.5 ± 4.1	0.43
LTI (kg/m^2^)	14.1 ± 3.0	13.7 ± 2.4	14.3 ± 3.3	0.51
FTI (kg/m^2^)	8.9 (6.4–13.0)	10.5 (8.3–12.8)	7.7 (5.9–13.3)	0.27

Data are presented as mean ± standard deviation or number (%)

*RKF* residual kidney function, *BP* blood pressure, *MAP* mean arterial pressure, *BMI* body mass index, *TBW* total body water, *ECW* extracellular water, *ICW* intracellular water, *LTI* lean tissue index, *FTI* fat tissue index

**Table 2:** Prescription of the type and number of peritoneal dialysis solutions

Type and number of exchanges	Total (*n* = 50)	Controlled BP group (*n* = 17)	Uncontrolled BP group (*n* = 33)	*P*-value
No. of daily 1.5% solution exchanges				0.76
0	37 (74)	11 (64.7)	26 (78.8)	
1	2 (4)	1 (5.9)	1 (3.0)	
2	6 (12)	3 (17.6)	3 (9.1)	
3	1 (2)	1 (5.9)	0	
4	4 (8)	1 (5.9)	3 (9.1)	
No. of daily 2.5% solution exchanges				0.75
0	5 (10)	1 (5.9)	4 (12.1)	
1	3 (6)	2 (11.8)	1 (3.0)	
2	9 (18)	3 (17.6)	6 (18.2)	
3	6 (12)	2 (11.8)	4 (12.1)	
4	27 (54)	9 (52.9)	18 (54.6)	
No. of daily icodextrin exchanges				0.29
0	39 (78)	15 (88.2)	24 (72.7)	
1	11 (22)	2 (11.8)	9 (27.3)	

Data are presented as number (%)

*BP* blood pressure

The uncontrolled BP group had a higher mean systolic BP (150 ± 12.5 mmHg vs. 116 ± 14.1 mmHg; *P* < 0.01) and mean diastolic BP (98.5 ± 9.4 mmHg vs. 76.5 ± 8.6 mmHg; *P* < 0.01).

**Fig. 1:**
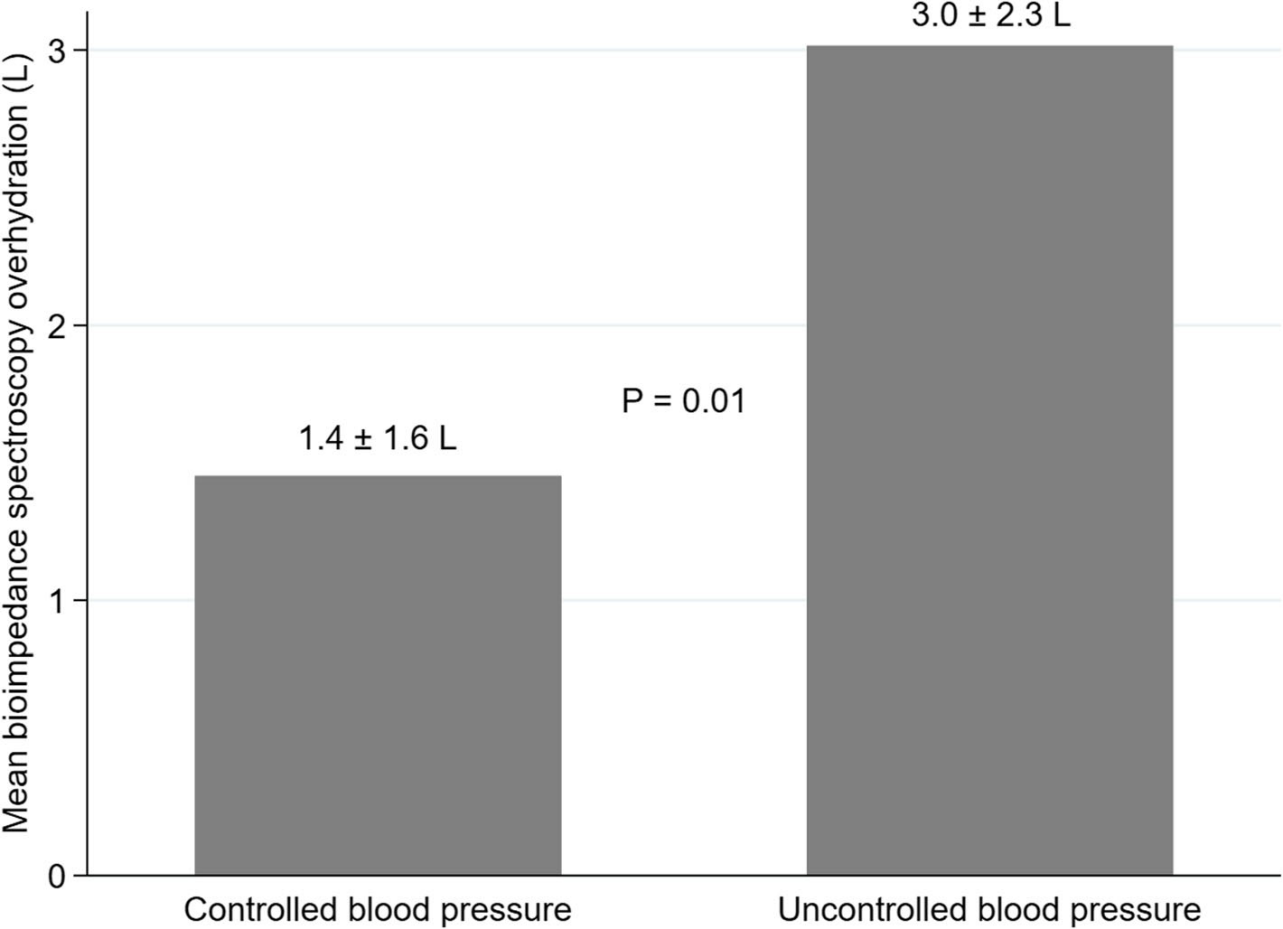
Bar graph of mean bioimpedance spectroscopy overhydration in controlled versus uncontrolled blood pressure groups

Regarding BIS measurements, there were no differences in TBW, ECW, ICW, ECW to TBW ratio, LTI or FTI. A statistically significant difference was found in OH between the controlled and uncontrolled BP groups (3.0 ± 2.3 L vs. 1.4 ± 1.6 L, respectively; *P* = 0.01) (Fig. 1).

**Fig. 2:**
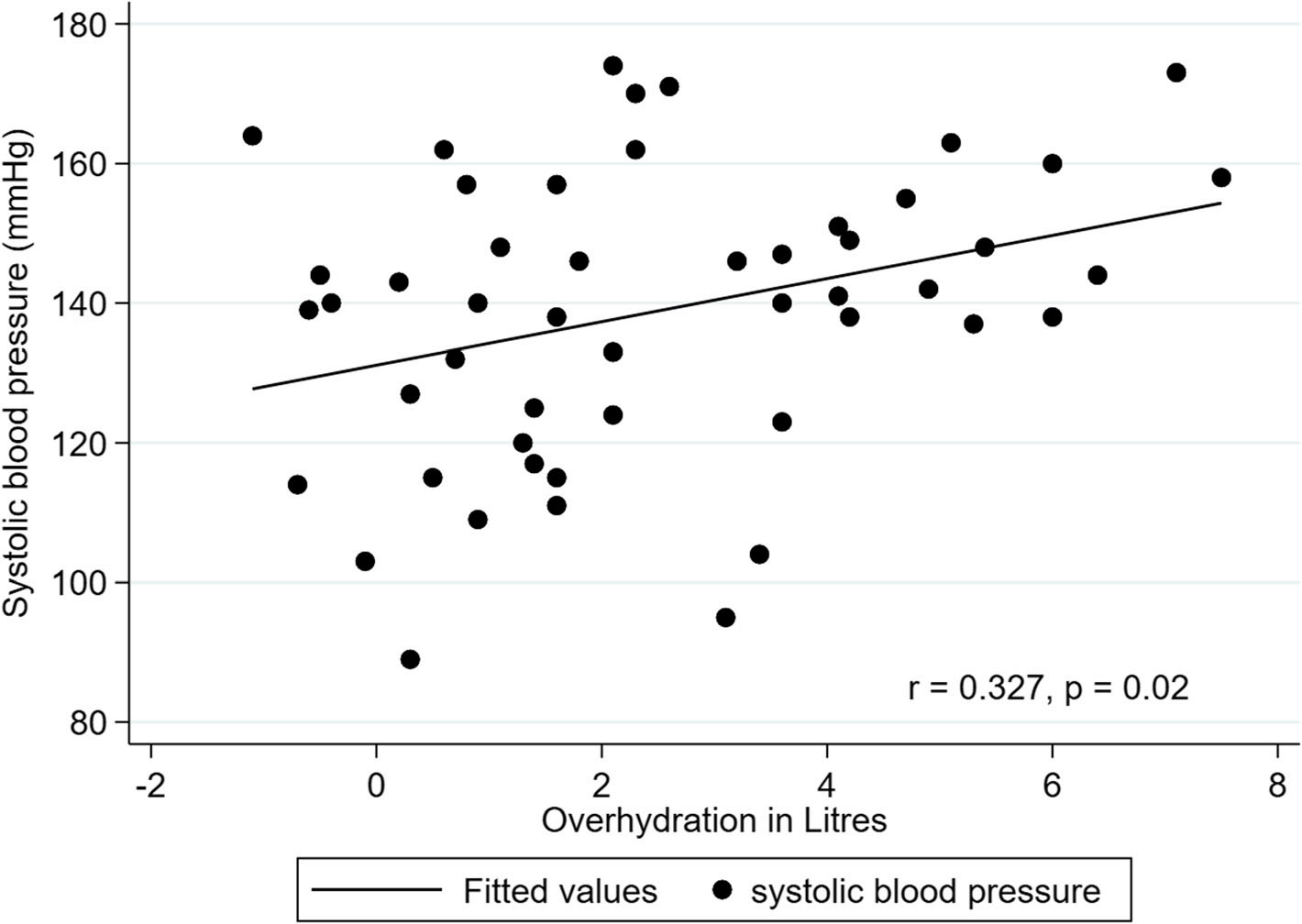
Relationship between overhydration (L) and systolic blood pressure (mmHg) with regression line (*r* = 0.327, *P* = 0.02)

**Fig. 3:**
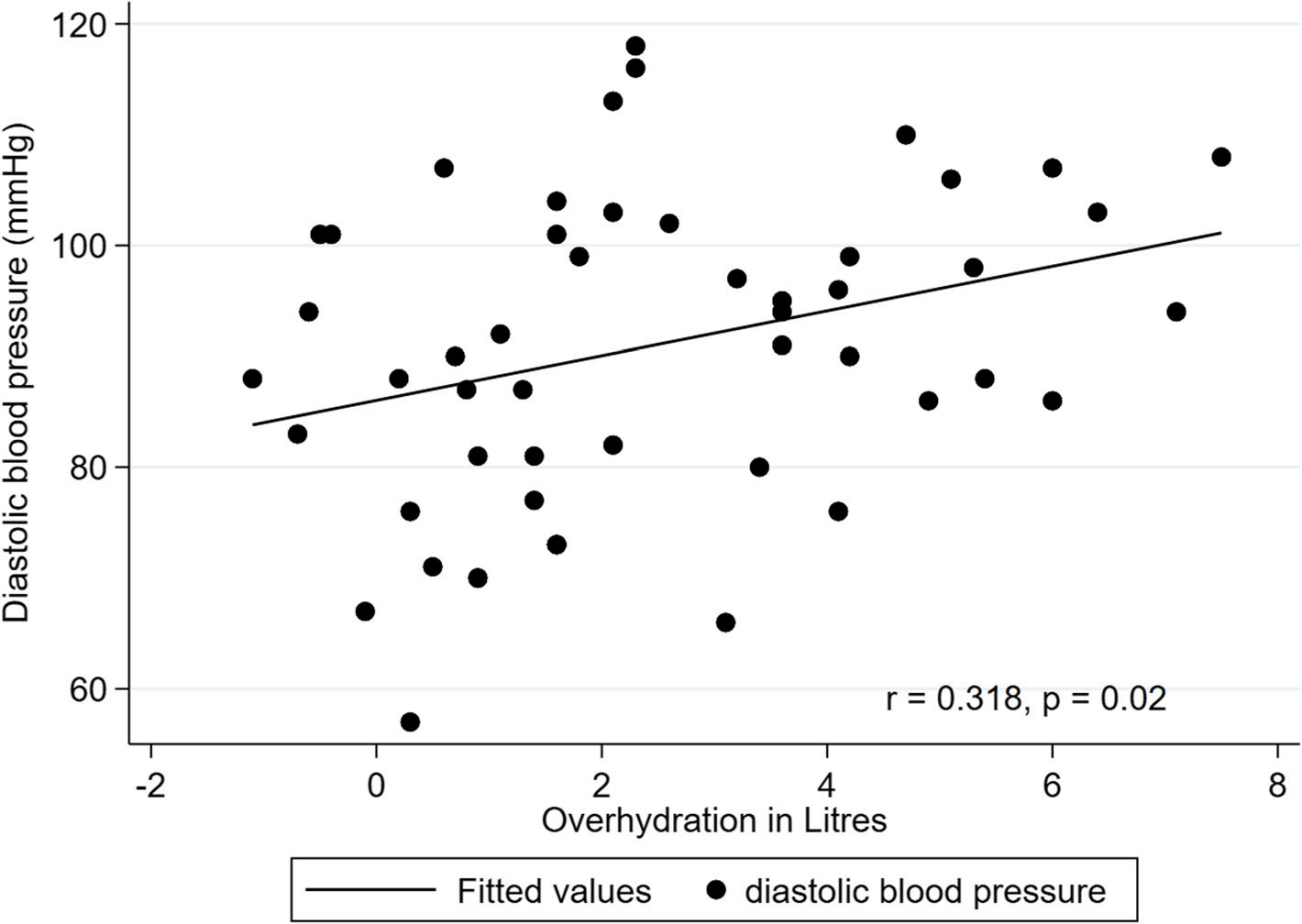
Relationship between overhydration (L) and diastolic blood pressure (mmHg) with regression line (*r* = 0.318, *P* = 0.02)

There were statistically significant correlations between OH and systolic BP (*r* = 0.327, *P* = 0.02) (Fig. 2), diastolic BP (*r* = 0.318, *P* = 0.02) (Fig. 3), mean arterial pressure (*r* = 0.338, *P* = 0.02), ECW (*r* = 0.557, *P* < 0.01) and ECW to TBW ratio (*r* = 0.474, *P* < 0.01); however, there were no significant correlations between OH and TBW (*r* = 0.229, *P* = 0.11), ICW (*r* = − 0.071, *P* = 0.63), dialysis vintage (*r* = − 0.220, *P* = 0.12), age (*r* = 0.02, *P* = 0.87), LTI (*r* = − 0.185, *P* = 0.21) or FTI (*r* = − 0.155, *P* = 0.29).

In a stepwise forward multivariable linear regression model, variables that predicted OH included mixed ancestry, presence of RKF, ECW and ECW to TBW ratio (Table 3).

**Table 3:** Multivariable linear regression model for overhydration

Variable	β	SE	t	95% CI	*P*-value
Age	−0.05	0.03	−1.75	−0.10 to 0.01	0.09
Mixed ancestry	1.05	0.45	2.35	−1.95 to − 0.15	0.02
RKF – Yes	−1.18	0.50	−2.34	−2.19 to −0.16	0.02
Number of anti-hypertensives	−0.10	0.22	−0.45	−0.55 to 0.35	0.66
ECW	0.45	0.08	6.00	0.30 to 0.60	< 0.01
ECW to TBW ratio	10.02	2.63	3.81	4.71 to 15.33	< 0.01

Model fit *r*^*2*^ = 0.597, adjusted *r*^*2*^ = 0.552

*SE* standard error, *CI* confidence intervals, *RKF* residual kidney function, *ECW* extracellular water, *TBW* total body water

## Discussion

We found that stable CAPD patients with uncontrolled BP had higher OH compared to patients whose BP was controlled. The reason for this finding is unclear. The uncontrolled BP group was older and therefore other causes of fluid overload such as undiagnosed heart disease may have contributed. Also, increased arterial stiffness may have been another factor contributing to the uncontrolled BP. A previous study reported a positive correlation between OH and arterial pulse wave velocity (PWV) [3], a measure of arterial stiffness. Another study found that age and fluid overload in a PD population were independent predictors of high PWV [21, 22]. Additional factors that may have contributed to poor BP control include poor dietary salt and water restriction, poor adherence to exchanges, malnutrition and hypoalbuminemia, systemic inflammation, and fast peritoneal transport status [1, 3, 23]. In the current study, although the prescription of 1.5 and 2.5% glucose dialysate solutions did not differ significantly, there may have been differences in the response to ultrafiltration between groups. A previous study reported that systolic BP was associated with faster peritoneal transport which may cause sodium retention [23].

Mixed ancestry and RKF were identified as predictors of OH using multivariable regression. In the Western Cape province of South Africa, nearly half the population is of mixed ancestral descent [24], therefore mixed ancestry may simply reflect the population demographic. Although there was no statistical difference between participants that reported having RKF, the paradoxical finding of RKF as a predictor of uncontrolled BP is probably due to our center’s PD first policy. With the limited availability of hemodialysis slots in our unit, most incident end-stage kidney disease patients accepted for kidney replacement therapy are offered CAPD as their initial dialysis modality. Since the dialysis vintage in the uncontrolled BP group was shorter, this group was more likely to report having RKF.

Although most of the patients that were prescribed icodextrin were in the uncontrolled BP group, the reason for its prescription was unrelated to poor BP or volume control. All these patients were either employed or were scholars. The use of icodextrin allowed them to avoid daytime exchanges during work/school hours. Sub-analysis of the uncontrolled BP group using icodextrin indicated that six of the nine patients had OH volumes ranging from 1.6 L to 5.1 L. We suspect that poor adherence to exchanges along with poor dietary restrictions may have contributed to this finding.

Although not statistically significant, FTI was higher in the normotensive group which may explain the higher BIS dry weight in this group since there were no differences in LTI. The higher body fat mass is probably related to the longer dialysis vintage in this group, which has been reported by others [25]. A subgroup analysis of PD patients stratified by dialysis vintage reported that lean tissue mass decreased over time while body fat mass increased [25]. With the longer and uninterrupted exposure of peritoneal membrane to the high glucose-containing dialysate solutions, the continuous absorption of glucose may have resulted in an increase in fat storage. Also, with the control of the uremic milieu, improvement in appetite with an increase in caloric intake, may have contributed to the higher fat mass.

Although no statistical difference in BIS dry weight was identified, it was lower in the uncontrolled BP group. Since we did not identify any differences in LTI or the ICW compartment between groups, we expected the volume of the ECW compartment in the uncontrolled BP group to be lower; however, there was a trend toward a higher ECW compartment in the uncontrolled BP group. This is supported by the findings of significant correlations as well as predictors on multivariable regression between OH and ECW, and OH and ECW to TBW ratio. Although the ECW compartment was expanded, there were very few clinical signs to suggest fluid overload. The documented clinically estimated dry weight was higher in the uncontrolled BP group and lower in the controlled BP group. Many studies have reported on the poor diagnostic accuracy of the clinical examination in identifying subclinical fluid overload [9–11]. Therefore, BIS may assist with a more objective measure of fluid status in stable CAPD patients.

This study would implicate that by improving OH through reducing dietary salt intake and the modification of and adherence to the PD prescription, better BP control may be achieved; however, a recent Kidney Disease Improving Global Outcomes Controversies Conference on BP and volume management in dialysis recommended that future investigations should focus on whether “bioimpedance-guided volume management will improve patient-centered and hard clinical outcomes” [26].

## Strengths and limitations

This was a small, single center study. Performing 24-h ambulatory BP monitoring may have resulted in more accurate BP measurements as well as eliminate the white coat effect; however, the use of office BP measurements was more pragmatic. Due to logistical constraints, we were unable to quantify ultrafiltration volumes and RKF.

## Conclusions

We found that stable CAPD patients with uncontrolled BP had higher OH compared to patients whose BP was controlled. Overhydration was confined to the extracellular water compartment. BIS may assist with a more objective assessment of dry weight, particularly in CAPD patients without obvious clinical signs of fluid overload.

## Data Availability

The datasets used and/or analysed during the current study are available from the corresponding author on reasonable request.

## Abbreviations

CAPD: Continuous ambulatory peritoneal dialysis
BIS: Bioimpedance spectroscopy
BP: Blood pressure
OH: Overhydration
IQR: Interquartile range
PD: Peritoneal dialysis
RKF: Residual kidney function
CANUSA: Canada-USA Peritoneal Dialysis Study Group
ADEMEX: Adequacy of Peritoneal Dialysis in Mexico
TBW: Total body water
ECW: Extracellular water
ICW: Intracellular water
LTI: Lean tissue index
FTI: Fat tissue index
TBH: Tygerberg Hospital
HREC: Health Research Ethics Committee
